# Immunoglobulin G4 in primary Sjögren’s syndrome and IgG4-related disease - connections and dissimilarities

**DOI:** 10.3389/fimmu.2024.1376723

**Published:** 2024-09-19

**Authors:** Maria Maslinska, Kinga Kostyra-Grabczak

**Affiliations:** Early Arthritis Clinic, National Institute of Geriatrics, Rheumatology and Rehabilitation, Warsaw, Poland

**Keywords:** immunoglobulin G4, Sjögren’s syndrome, IgG4-related disease, autoimmunity, lymphomas

## Abstract

Primary Sjögren’s syndrome (pSS) is an autoimmune disease, with B cell hyperactivation and autoantibody production as its immunological hallmarks. Although the distinction between immunoglobulin G4-related disease (IgG4-RD) and pSS, based on the presence or absence of certain autoantibodies, seems easy to make, possibility of elevated serum IgG4 concentration and often similar organ involvement may lead to a misdiagnosis. The increased serum concentration of IgG4 in IgG4-RD is not clearly linked to the pathogenesis of IgG-RD and it has been suggested that it may constitute just an epiphenomenon. The aim of this article is to discuss the presence of IgG4 in pSS and IgG4-RD and its potential significance for these two diseases.

## Introduction

Primary Sjögren’s syndrome (pSS) is a chronic autoimmune disease with a dominance of immunological features such as hypergammaglobulinemia, B and T cell activation, autoantibody production mainly against ribonucleoproteins (anti-SSA/Ro and anti-SSB/La antibodies), low levels of C3 and C4 components of the complement system, rheumatoid factor (RF) and cryoglobulin production. The main autoimmune and inflammatory process, with infiltrations of mononuclear cells, takes place in exocrine glands and internal organs. Due to the tropism of exocrine glands, of lacrimal and salivary glands in particular, the dysfunction of those glands is observed, causing dryness - one of the main clinical features of pSS (1).

The presence of anti-Ro/SSA and anti-La/SSB is typical for pSS (1). Less often, immunological tests may reveal anti-centromere B (CENP-B) and anti-citrullinated protein antibodies (ACPA) or antibodies more specific to other connective tissue diseases (2, 3). Rheumatoid factor may also be present in IgM, IgA, and IgG classes of immunoglobulins (4, 5). The association of pSS and primary biliary cholangitis with AMA-M2 autoantibodies or autoimmune thyroiditis with thyroid peroxidase antibodies (TPOAb) and thyroglobulin antibodies (TgAb) is also well established (6, 7).

Immunoglobulin G4-related disease (IgG4-RD) is another autoimmune disorder, which shares some of its clinical symptoms with pSS, including the presence of the inflammatory and autoimmune process in the salivary and thyroid glands and other similar organs. Its identification underlines the attention paid to immunoglobulin G4 in recent years, its role in the human immune system, and the connection of this antibody to various clinical states.

Interestingly, for years, Mikulicz’s disease (MD)—currently recognized as a clinical manifestation of IgG4-RD—was considered a form of pSS. It was described by a Polish surgeon, Jan Mikulicz-Radecki, in 1888 and its symptoms include sialadenitis and dacryoadenitis with salivary and lachrymal gland enlargement (8, 9). Another example of isolated submandibular gland involvement is chronic sclerosing sialadenitis, known as Kutner’s tumor (chronic sclerosing sialadenitis), which is also currently classified as a manifestation of IgG4-RD. It occurs without the involvement of other salivary glands, which is one of the elements that distinguishes it from pSS, where the characteristic feature is the involvement of the parotid and submandibular glands (8). The increased serum concentration of IgG4 is a recognized immunological determinant of IgG4-RD, while in the case of pSS, various relationships are presented - both a decrease and, in some studies, an increase in the concentration of this immunoglobulin. The still not fully recognized pathophysiological role of the IgG4, the research, that led to the distinguishing of IgG4-RD, as well as the similarities between this disease and pSS were the inspiration to write the present article.

## Overview of the unique role of IgG4

In human serum, the IgG4 class of immunoglobulins is least abundant among other immunoglobulins G and constitutes approximately 5% of IgG (10). Serum IgG4 levels increase gradually from birth and by the age of approximately 10 years they usually reach adult levels, hindering the assessment of IgG4 levels in young children (11). Isolated IgG4 deficiency is very rare and more often occurs in combination with a deficiency of other immunoglobulins (IgG1, IgG2, or IgA). There are observations (mainly in children) indicating that in a case of IgG4 deficiency, recurrent respiratory infections, allergies, candidiasis, and chronic diarrhea occur (12, 13). IgG4 deficiency was also observed in patients with associated inflammatory bowel disease (14). Currently, a lot of attention is being paid to IgG4 hypergammaglobulinemia, which may occur in a healthy population (in ~5%), although increased serum concentration in a healthy population has not yet been proven to have clinical consequences (15).

IgG4 is attributed with an anti-inflammatory role (e.g., response to parasitic infections and allergies) and is also associated with a potentially pathogenic role in autoimmune diseases and with a response to biological treatment or cancer development (16).

Such varied perceptions of the role of IgG4 in immune processes are related to its specific features and abilities, different from other IgG molecules.

In 1997, the structure of an IgG4 Fc fragment was described (17, 18). The structure of human IgG4 is highly homologous (over 90%) to other IgG subclasses, but its unique properties result from specific amino acid differences in the heavy chain, especially in the hinge region, which allows for the phenomenon of fab arm exchange (FAE) (observed *in vitro* only for this subclass of immunoglobulin G) as well as the variability in CH3 domains (19, 20).

The unique property of IgG4 is FAE, which is possible due to relatively labile disulfide bonds between the heavy chains of IgG4 molecules, which allow for the phenomenon of recombination (exchange of half-molecules, each consisting of one heavy chain and one light chain). Such an exchange between two IgG4 molecules allows for the formation of a monovalent (for each antigen)/bispecific antibody, contrary to other IgG monomers, which are bivalent, but monospecific in nature (21, 22). The FAE phenomenon is also favored by a weaker CH3-CH3 interaction in IgG4, caused by the replacement of lysine with arginine (position 409) (19).

In a physiological state, IgG4 is an anti-inflammatory antibody and weakly activates the complement system. Under conditions of increased IgG4 concentration, the consumption of complement components was observed, which indicates such a possibility. It is suggested that special properties of IgG4, such as FAE and glycosylation of Fc fragments, affect complement activation (10, 19). It is also proposed that IgG4 blocks the binding of other immunoglobulin subclasses to the complement component 1q (C1q).

The glycosylation of the Fc IgG4 fragment is another process that plays a role in altering IgG4 effector functions (23). Glycosylation changes the affinity of IgG4 to the different FcγRs (24). Studies on the glycosylation of IgG and IgG4, including in the pathophysiology, (e.g., IgG4 in primary membranous nephropathy causes activation of the lectin pathway and induction of podocyte damage) indicated a pro-inflammatory role of this process and the possibility of the complement activation also by glycoforms of IgG4 (25, 26). Current research indicates that the process of glycosylation of IgG, including IgG4, may have a pro-inflammatory role and is associated with the pathogenesis of various inflammatory diseases. However, the assessment of this phenomenon requires further research.

In Oskam N et al. (27), the authors showed that complement activation by IgG4 is possible, but only at high antigen densities and high antibody concentrations. The researchers managed to demonstrate that such an activation occurred only through the classical pathway. It is possible that these potential pathways are enhanced by galactosylation, while the occurrence of FAE has the opposite effect. However, these findings do not explain the pathogenic role of IgG4 in IgG4-RD or, for example, in primary membranous nephropathy (pMN).

The antigen-specific activity of IgG4 under the influence of chronic antigen stimulation in allergic diseases and in the course of immunotherapy was studied more widely and earlier than the problem of IgG4-related disease (16).

In allergic diseases, as well as in IgG4-RD, T helper type 2 (Th2) cells play an important role associated with interleukin 4 (IL-4) and 13 (IL-13) production (Th2 cytokines). These cytokines stimulate B cells to switch to class IgG4 immunoglobulin production (28).

Because of this stimulation, B cells exposed to an allergen, in addition to producing allergenic IgE, produce IgG4. Immunoglobulin G4 competes with IgE to prevent the activation and the subsequent degranulation of mast cells and basophils. Subsequently, the IgE to IgG4 (IgE/IgG4) ratio decreases. This situation also occurs during allergen-specific immunotherapy. In such circumstances, the persistence of increased IgG4 concentration may be observed for a longer period of time, even up to 3 years (29). Because of the described observations in some diseases with IgE elevation, the protective role of IgG4 has been suggested.

A study on anti-drug antibodies in the biological treatment of RA revealed that adalimumab-treated patients developed antibodies against adalimumab mainly in the IgG4 isotype, rather than in the IgG1 isotype (30). Such observations were associated with longer (3 years in cited study) drug exposition. It was demonstrated that a longer exposition to an antigen (drug) can cause the development of more IgG4 anti-drug-antibodies—a finding which corresponds with other research on time of exposure to allergen/antigen and IgG4 antibodies production.

In summary, it is believed that in the case of allergic diseases and immunotherapy, IgG4 may have a potentially protective effect. A similar increase of both IgG4 and IgE levels is observed in helminth infections (31, 32). Furthermore, beekeepers and laboratory workers may exhibit high serum levels of the allergen-specific IgG4, which protects them from an anaphylactic reaction (20).

However, it is still not clearly established whether increased serum concentration of IgG4 plays an important role in diseases such as IgG4-RD or Crohn’s disease (CD) or whether it should be considered an epiphenomenon only (33).

## IgG4 detection

In clinical practice, the detection of IgG4 currently is based on different tests, namely, radioimmunoassay (RIA), enzyme-linked immunosorbent assay (ELISA), and immunonephelometry, with two latter ones being most commonly used. In Su et al. (34), a comparison study between these tests proved good compatibility between ELISA and the nephelometric assays. The fact that the costs of performing an ELISA test are lower than immunonephelometry may also be important in planning research.

An antigen-binding radioimmunoassay (RIA) was used particularly in the oldest studies, in which it was used to measure liquid-phase IgG4 antibodies (35).

For years, the problem of establishing a cut-off value for a significant IgG4 concentration, in a way as to maintain the greatest sensitivity and specificity of the tests, has been discussed. Especially since the identification of IgG4-RD, it has become important to determine what value of IgG4 concentration is most likely to be associated with this disease. The classification criteria for IgG4 – RD adopted a value of 1.35 (135 mg/dL) as the cut-off value (36) but in some studies, higher levels were noted as being more specific and sensitive (37, 38).

Carruthers et al. (39) noted that elevated serum IgG4 concentrations had only 60% specificity and a 34% positive predictive value (PPV). Therefore, the authors suggest that a doubled cut-off value may increase the specificity of the test.

In some studies, researchers highlighted that elevated serum concentrations of IgG4 or plasmablasts may be a hallmark of IgG4-RD (40).

This approach is determined by the difficulties in interpreting symptoms and diagnosing IgG4-RD in the presence of a normal/low serum IgG4 concentration. Although such a discussion is currently taking place mainly in relation to IgG4-RD, its outcome may have further consequences, leading to a re-evaluation of diagnostic criteria for other diseases, including pSS, related in various ways to IgG4.

## The outline of pSS pathogenesis

The discussion of the potential role of IgG4 in pSS requires a basic understanding of pSS pathogenesis—a problem that still remains not yet fully understood. However, certain factors, such as a breach of immune tolerance, endothelial damage, release of autoantigens, and B cell activation with autoantibody production, were confirmed as immunological features of this chronic autoimmune disease, which is also known under the name of “autoimmune epithelitis” (41).

In recent years, the role of the activity of the interferon pathway in pSS, as well as other autoimmune rheumatic diseases, has been examined. The overexpression of type I IFN is called IFN-I signature (42). Other studies highlighted the significance of T lymphocytes as cells that influence the activation of B lymphocytes with the participation of stimulating factors (CTLA-4) and through the production of B cell stimulating factors such as BAFF/BlySS (43).

Sicca symptoms due to exocrine gland involvement remain the main clinical feature of pSS, although it does not take place in the early stages of pSS. Importantly, in different age groups of patients, we can expect different symptoms as they present with different levels of immune system activity and varied degrees of organ and exocrine gland damage. Several pSS phenotypes have been described and the type designation highlights sicca symptoms, systemic involvement, and organ damage as follows: sicca without organ damage, sicca with glandular involvement/mild systemic involvement, and sicca with severe systemic involvement (44).

The main immunological hallmarks of pSS are anti-ribonucleoprotein antibodies (anti Ro/SSA and anti-La/SSB). They belong to antibodies against extractable nuclear antigens (ENA) which are present in the cell’s cytoplasm. Anti-Ro/SSA ab was divided in 1981 by Lerner et al. (45) in two subclasses depending of a molecular weight: anti-Ro52 kDa and anti-Ro60 kDa.

Interestingly, Ro52 and Ro60 antigens are coded on different chromosomes. Ro60 is coded on chromosome 19 and forms complexes with small cytoplasmic RNA (hY-RNA complexes) which may be involved in binding misfolded mRNA and influence its degradation.

Ro52 is coded on chromosome 11, belongs to the tripartite motif protein (TRIM) family, and is involved in inflammation and apoptosis (46).

Some researchers suggest that anti-Ro60 abs are sufficient to diagnose pSS, while anti-La/SSB abs are heterogenous and may be present in other autoimmune diseases, such as systemic lupus erythematosus (SLE), neonatal lupus erythematosus (NLE), and inflammatory myositis as well as pSS. As has been concluded in recent studies, anti-Ro60 abs are considered a triggering factor for autoimmunity (glomerulonephritis, ultraviolet light radiation sensitivity). Anti-Ro52 abs are associated also with other autoimmune diseases, such as systemic sclerosis, rheumatoid arthritis, and primary cholangitis.

However, despite differences between anti-Ro/SSA subclasses, the measurement of anti-Ro/SS-A antibodies as a complex in an ENA panel is sufficient for the determination of their presence for diagnosis according to the current classification criteria of pSS (46).

**Figure 1:**
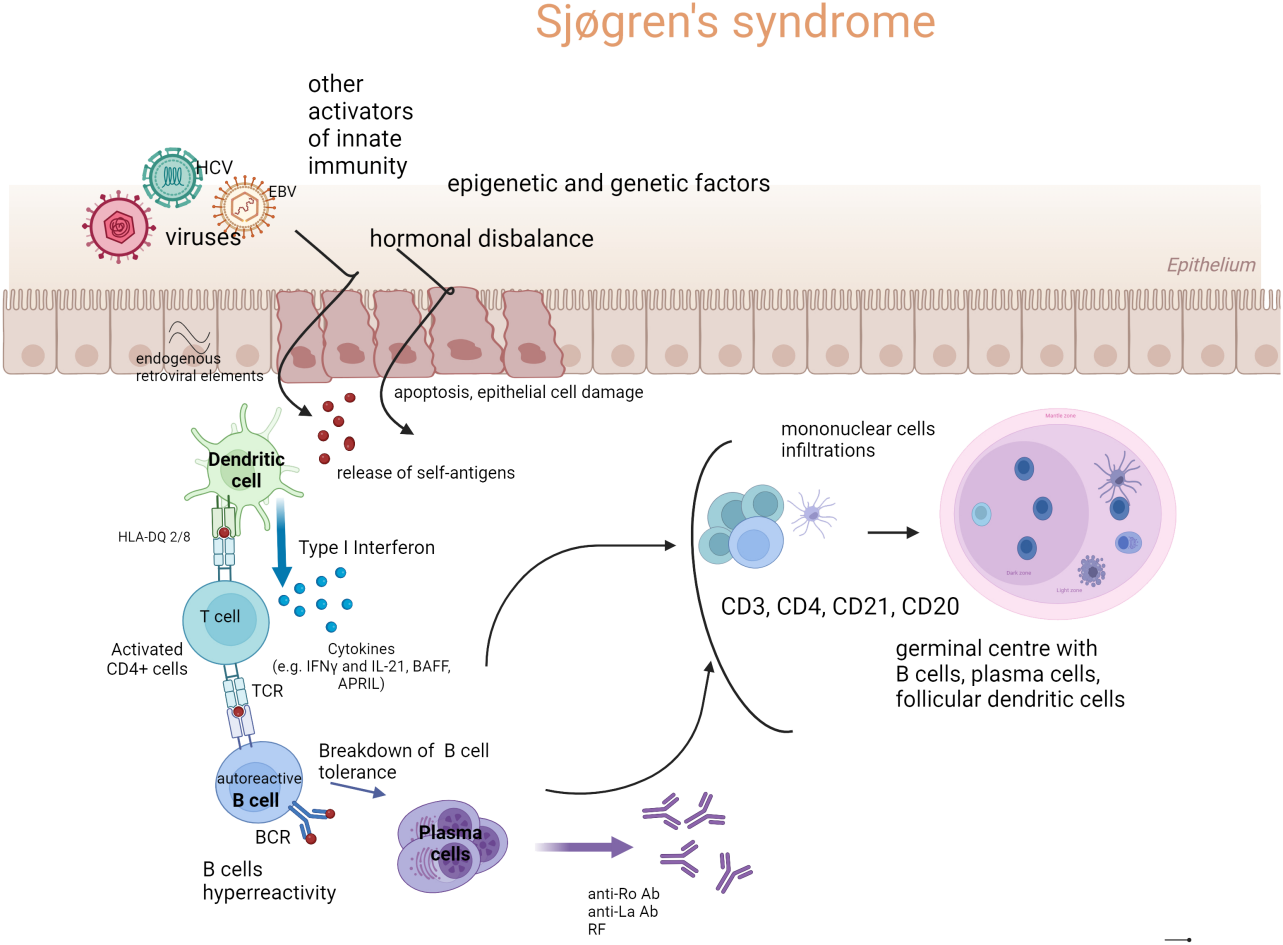
The outline of Sjögren’s syndrome pathogenesis (Created with BioRender.com). Genetic susceptibility, environmental factors such as mainly viral infections, but also bacterial infections, dysbiosis (microbiome disbalance), endogenous retroviral elements, hormonal disbalance (estrogen deficiency), and ultraviolet radiation (UV) are involved in epithelial cell damage and apoptosis. The endothelial cell damage and release of autoantigens stimulate innate and acquired immunity. The first-line immune response leads to the activation of dendritic cells (DC), especially of a type I signature of the IFN pathway, which releases interferons (IFNs). Subsequently, cross-talk between DC and T cells and their production of B cell stimulating factors (e.g., IFNγ, B cell activating factor (BAFF), a proliferation-inducing ligand (APRIL), and interleukin-21 (IL-21)) cause the activation of B cells and their hyperreactivity. Newly formed plasma cells produce autoantibodies, to ribonucleoproteins in particular, such as anti-Ro/anti-La antibodies and rheumatoid factors (RF) in various classes of immunoglobulins. In target organs (primarily salivary glands), cell infiltrates are formed, consisting of mononuclear cells such as dendritic cells (mainly CD21), T lymphocytes (CD4+, CD3+), CD20 B lymphocytes, and plasma cells. Under the influence of chronic and intense stimulation, tertiary lymphatic structures may be formed—germinal centers (GC), and activation of B cells beyond the control of T lymphocytes may occur. Such stimulation carries the risk of developing a lymphoproliferative process in pSS.

In the **Figure 1**, the outline of the recognized pSS pathogenesis is presented.

## Diagnosis of primary Sjögren’s syndrome

To confirm pSS diagnosis, the European League Against Rheumatism/American College of Rheumatology (EULAR/ACR) classification criteria should be met, and a differential diagnosis should also be performed to exclude other diseases (47). The elements of pSS classification criteria are presented in **Table 1**.

**Table 1:** EULAR/ACR classification criteria for primary Sjogren’s syndrome diagnosis (47).

Domain	Parameter	Points	Exclusions
Mouth dryness	Unstimulated salivary flow	1	Active HCV (PCR), confirmed sarcoidosis, GvHD, neck and head radiotherapy, amyloidosis, IgG4-RD, previous lymphoma.
Eye dryness	Schirmer’s test ≤ 5mm/5 min	1	
	Ocular Staining Score (OSS) ≥5 or Bijsterveld’s test ≥4	1	
Immunological	Anti-SSA/Ro antibodies	2	
Histopathological	*FS* ≥ 1	2	

To fulfill the classification criteria, the total requested score must be ≥4.

OSS, ocular staining score (48); HCV, hepatitis C virus; GvHD, growth versus host disease; FS, focus score (1 for each mononuclear cell infiltrate, which contains at least 50 inflammatory cells, that is present in a 4 mm^2^ of a biopsy section).

The classification criteria are met if the final scoring reaches a minimum of four points and the exclusion criteria are not fulfilled.

## A short introduction to IgG4-related disease

IgG4-related disease is a chronic autoinflammatory disease of an unknown etiology. The principal symptoms of IgG4-RD include the elevation of IgG4 serum concentration, characteristic infiltrations of mononuclear cells, fibrosis, and formation of pseudotumors. C-reactive protein elevation and fever are not characteristic of IgG4-RD as for other autoimmune-autoinflammatory diseases, e.g., Castleman’s disease, antineutrophil cytoplasmic antibody-associated vasculitis (especially eosinophilic granulomatosis with polyangiitis EGPA)—a phenomenon which should be considered in a differential diagnosis.

A histopathological examination is considered the gold standard of IgG-RD diagnosis, revealing storiform fibrosis, inflammation with infiltration by lymphocyte and plasmatic cells, eosinophilia, and obliterative phlebitis. The formation of germinal centers and lymphoid follicles is also observed.

A storiform pattern of fibrosis (similar to the woven fabric pattern in microscopy assessment) is considered typical for IgG4-RD, however, it can also be observed in neoplastic changes. In the late stage of IgG4-RD, acellular fibrosis dominates (49). Storiform fibrosis is accompanied by dense lymphoplasmacytic infiltrates, often partially eosinophilic infiltrates and obliterative phlebitis.

Obliterative vascular disease is a unique feature of IgG4-RD, not observed in other vasculitis, e.g., granulomatosis with polyangiitis (GPA), polyarteritis nodosa (PN), or microscopic polyangiitis (MPA). In IgG4-RD, vessel wall necrosis is not observed (49).

In infiltrations, IgG4 + plasma cells are dominant (greater than 10 IgG4 + plasma cells/HPF) with a ratio of IgG4 +/IgG + cells greater than 40%.

The current classification criteria for IgG4 - RD diagnosis were established and published in 2019 (36). The verification of whether a patient meets the classification criteria of IgG4-RD is divided into three steps.

The first step is based on the entry criteria (**Table 2**).

**Table 2:** Entry criteria for the evaluation for IgG4–RD according to ACR/EULAR classification criteria.

The involvement of organs characteristic/typical in IgG4-RD
Pancreas, salivary glands, bile ducts, orbits, kidney, lung, aorta, retroperitoneum, pachymeninges, and thyroid gland (only Riedel's thyroiditis), as well as evidence of an inflammatory process accompanied by a lymphoplasmacytic infiltrate in one of the listed organs, which cannot be attributed to any other condition.

The second step is only possible after meeting the entry criteria. It requires a consideration of possible exclusions, which are listed in **Table 3**.

**Table 3:** List of exclusions (36).

Exclusions
**1. Clinical** Documented recurrent fever >38°C (in the absence of any clinical features of infection). No objective response to glucocorticoids [no response to a treatment with prednisone at a minimum of 40 mg/day (~0.6 mg/kg/day) for 4 weeks]. The decrease of IgG4 serum concentration only is not considered as a response to treatment. **2. Serological** Leukopenia and thrombocytopenia without alternative explanation* Peripheral eosinophilia >3,000 mm3. Presence of antineutrophil cytoplasmic antibodies (ANCA)** Presence of AID antibodies (anti-dsDNA, RNP, SM antibodies)*** Cryoglobulinemia with another explanation for a patient’s condition Other AID-specific antibodies**** **3. Radiological** Rapid radiological progression Long bone abnormalities typical of Erdheim-Chester disease Splenomegaly Radiological findings typical or suspected of infection or malignancy **4. Histopathological findings:** Cellular infiltrates suggesting malignancy that has not been sufficiently evaluated Markers consistent with inflammatory myofibroblastic tumour (IMT)***** Dominant neutrophilic inflammation Necrotizing vasculitis Prominent necrosis Primarily granulomatous inflammation Pathologic features of macrophage/histiocytic disorder (hemophagocytic lymphohistiocytosis spectrum diseases, HLH)****** **5. Diagnosis with another disease:** Multicentric Castleman's disease Crohn's disease******* Ulcerative colitis******* Hashimoto thyroiditis********

ANCA, antineutrophil cytoplasmic antibody; dsDNA, double-stranded DNA; IgG4-RD, IgG4-related disease; MPO, myeloperoxidase; PR3, proteinase 3; anti-SM antibody, anti-Smith antibody.

*Hematologic features atypical for IgG4-RD. **Antibodies against proteinase 3 or myeloperoxidase. ***Autoantibodies of low specificity such as RF and ANA do not account for an exclusion and are not clear exclusions.

****Anti-Ro/SSA, anti-La/SSB, double-stranded DNA, RNP, and Sm antibodies in titers greater than normal suggest an alternative diagnosis. Other autoantibodies associated with a high specificity for another immune-mediated condition suggest another diagnosis. Such specific autoantibodies include anti-synthetase antibodies (e.g., anti–Jo-1), anti–topoisomerase III (Scl-70), and anti–phospholipase A2 receptor antibodies.

****IMT benign tumor consisted with myofibroblastic and fibroblastic cells and lymphocytes and eosinophils.

*****HLH haemophagocytic lymphohistiocytosis this is a hyper-inflammatory disease with multiorgan failure, primary (genetic mutations), secondary (triggering by infection, malignancy, autoimmune disorders).

******If pancreato-biliary disease is present. *******If the thyroid is the only proposed location of disease manifestation.

In the final step, verification of the inclusion criteria is performed. Inclusion criteria are divided into eight domains. The findings in each domain are weighted, and cases with 20 points or more are classified as IgG4-RD. However, according to the ACR/EULAR IgG4 classification criteria, if any exclusion criterium is met, the patient cannot be further considered as having IgG4 -RD. As a rule, the highest weighted item in each domain is scored (**Table 4**).

**Table 4:** Inclusion domains and items included in the IgG4 ACR/EULAR classification criteria (36).

Histology	Uninformative biopsy Dense lymphocytic infiltrate Dense lymphocytic infiltrate and obliterative phlebitis Dense lymphocytic infiltrate and storiform fibrosis with or without obliterative phlebitis	0 4 6 13
Immunostaining	IgG4+:IgG+ ratio is 0%–40% or indeterminate and the number of IgG4 + cells/hpf is 0–9. The IgG4+:IgG+ ratio is ≥41% and the number of IgG4 +cells/hpf is 0–9 or indeterminate or (2) the IgG4 +:IgG+ ratio is 0–40% or indeterminate and the number of IgG4+ cells/hpf is ≥10 or indeterminate. IgG4+:IgG+ ratio is 41%–70% and the number of IgG4 + cells/hpf is ≥10 or the IgG4+:IgG+ ratio is ≥71% and the number of IgG4 + cells/hpf is 10–50. IgG4+:IgG+ ratio is ≥71% and the number of IgG4 + cells/hpf is ≥51	0 7 14 16
IgG4 serum concentration	Normal or not checked >Normal but <2× upper limit of normal 2–5× upper limit of normal ≥>5× upper limit of normal	0 4 6 11
Lacrimal, parotid, sublingual and submandibular glands (bilateral)	No set of glands involved One set of glands involved Two or more sets of glands involved	0 6 14
Chest	Not checked or neither of the items listed is present Peribronchovascular and septal thickening Paravertebral band-like soft tissue in the thorax	0 4 10
Pancreas and biliary tree	Not checked or none of the items are present Diffuse pancreas enlargement (loss of lobulations) Diffuse pancreas enlargement and capsule-like rim with decreased enhancement Pancreas (either of the above) and biliary tree involvement	0 8 11 19
Kidney	Not checked or none of the items listed are present Hypocomplementemia Renal pelvis thickening/soft tissue Bilateral renal cortex low-density areas	0 6 8 10
Retroperitoneum	Not checked or neither of the items listed is present Diffuse thickening of the abdominal aortic wall Circumferential or anterolateral soft tissue around the infrarenal aorta or iliac arteries	0 4 8

hpf/HPF, high-power field.

Additional notes: a) in the immunostaining domain biopsies from lymph nodes, mucosal surfaces of the gastrointestinal tract and skin are not acceptable; b) ‘Indeterminate’ means that a pathologist is unable to clearly quantify the number of positively stained cells within an infiltrate, yet can still ascertain that the number of cells is at least 10/hpf.

The introduction of the 2019 ACR/EULAR IgG4-RD criteria is an attempt to exclude—with the optimal precision in the diagnosis of this multiorgan and multisymptomatic disease—all mimickers and maintain sufficient sensitivity and specificity (the first validation cohort had a specificity of 99.2% and sensitivity of 85.5%, and the second validation a specificity of 97.8% and sensitivity of 82.0%) (36).

## Antibodies in the IgG4 subclass

In clinical practice, adverse reactions to food ingredients manifest with symptoms of intolerance as well as with the production of specific antibodies in various classes of immunoglobulins, especially in IgE, IgG, or IgG4. In food intolerance, IgG4 antibodies are associated with basophils and with mastocyte degranulation—elements similarly active in other allergic reactions. In food intolerance, total IgG and IgG4 are significantly increased. While an initial elevation of IgE is observed, a subsequent increase in IgG/IgG4 ratio constitutes a delayed persistent phase (from 24 hours to 5 days) of food intolerance (50). Therefore, for food allergy, diagnostic ELISA tests have been developed for specific IgG4 antibodies against food antigens (50).

In rheumatoid arthritis (RA), specific anti-citrullinated cyclic peptide antibodies (ACPAs) in the IgG4 class of immunoglobulins may occur. Carbone et al. (51) presented a metanalysis from three studies on 328 RA patients in total and concluded that elevated IgG4 ACPA was observed in 35.98% of patients. The Fab segments of RF can react with the Fc part of the IgG molecule and mainly generate IgM(RF)-IgG immune complexes. They can also recognize the Fc domains of IgG4 to form IgG4-RF immune complexes which may activate the complement system and cause synovial injury (52). In patients with RA, increased IgG4 serum concentration was also observed as was the presence of IgG4 in the inflammatory active synovium.

The focus on IgG4 led to the search for connections between this immunoglobulin and other autoimmune diseases. It seems obvious that, especially in IgG4-RD, various IgG4 associations were analyzed. Kiyama et al. (53) showed that in spite of the elevation of IgG4 in the sera of patients with IgG4-RD, the production of antinuclear antibodies (ANA) antibodies in the IgG4 subclass was not observed. Therefore, the authors suggested that the elevation of IgG4 levels is non-specific (epiphenomenon?) and not pathogenetic, concluding that IgG4-RD is not an autoimmune disease.

Demirci et al. (54) found a significant increase in serum levels of IgG4 in patients with celiac disease (CD) versus the control group and the authors concluded that IgG4 levels can constitute a biochemical marker for CD.

In an animal model in the study by Bi et al. (55), it was found that switching anti – ADAMTS13 (a disintegrin and metalloproteinase with a thrombospondin type 1 motif, member 13) to the IgG4 subclass had a protective effect in a IgG4-mediated disease, thrombotic thrombocytopenic purpura (TTP), in contrast to IgG3 anti-ADAMTS13 antibodies. In pemphigus foliaceus (PF), autoantibodies to desmoglein 1 (anti-Dsg1) in the IgG4 subclass of immunoglobulins played an opposite role by reducing FcγR-binding affinity or ablating FcγRs, which enhanced their pathogenic function. In PF, the IgG1 subclass was revealed to be non-pathogenic (56).

An interesting topic, however beyond the scope of this article, is the existence of IgG4-nervous system dependent diseases including myasthenia with muscle specific tyrosine kinase (MuSK) antibodies or chronic inflammatory demyelinating polyneuropathy (CIDP) with neurofascin-155, contactin-1/CASPR-1 antibodies, and anti- LGI 1 as well as in CASPR2-associated limbic encephalitis, neuromyotonia, and Morvan syndrome (57, 58). The cases of anti-IgLON5 and anti-DPPX-spectrum CNS diseases related to IgG4 were also described (59).

## Sjögren’s syndrome and antibodies in the immunoglobulin G4 class of immunoglobulins

Primary Sjögren’s syndrome is relatively common among systemic diseases of a connective tissue and is associated with the hyperactivity of B lymphocytes, which increases the possibility of the emergence of autoantibodies.

Antibodies to ribonucleoproteins are characteristic of pSS, but only anti-Ro/SS-A antibodies are included in the current classification criteria for this disease (47). However, antinuclear antibodies are present in the majority of pSS patients. Kiyjama et al.’s work (53) shows that ANA-IgG4 antibodies are rare in rheumatic diseases. Among the patients analyzed in the study only one, with pSS, had ANA-IgG4 antibodies. This patient did not meet the criteria for IgG4-RD. Although he had anti-Ro/Ss-A antibodies, the researchers did not indicate whether those included anti-Ro-IgG4. Antinuclear antibodies are usually present in IgG1-3 subclasses. They can also be present in low titers in IgG4-RD (a higher titer constitutes an exclusion criterion in IgG-RD diagnosis).

Wahren and colleagues (60), in 1994, investigated humoral response to Ro60kDa/SS-A and La/SS-B antigens and found that IgM and IgG (1-4) responses to these antigens coexist, while among IgG antibodies, IgG1 dominates.

In Sjögren’s syndrome, polyclonal hypergammaglobulinemia is one of the main laboratory findings and immunoglobulins usually belong to various classes. In pSS, hyperglobulinemia is associated with high serum levels of RF (IgM, IgA, and IgG). In rare cases, immunoglobulins of one class are responsible for the hyperglobulinemia. Liu et al. (61) studied immunoglobulin profiles in pSS and SLE patients. They showed that in pSS, IgG1, IgG2, and IgG3 were usually increased among the subclasses of immunoglobulin G, while there was a visible and significant reduced level of IgG4 in the sera of pSS patients and a decrease of the IgG4 to IgG ratio. Interestingly, in a cited work, the serum concentrations of IgG1-3 immunoglobulins were similar in pSS and SLE patients, however, a difference was observed between pSS and SLE in the IgG4 concentration. The authors did not find an explanation for this fact. In another work, a lower serum concentration of IgG4 in the pSS group (p=0.0435) and a significantly lower (p=0.0035) ratio of serum IgG4 to total IgG compared to healthy subjects, were confirmed. This study also showed a weak negative correlation (r= - 0.274) between C4 component complement levels and IgG4 (62).

There are also studies of pSS patients in which an increase of Ig-G4 serum concentration was shown, but it should be taken into account that such results concerned a small number of pSS patients, only 7.5% percent (n=10) of the pSS group (63).

## The role of IgG4 in a context of the development of lymphomas and sialadenitis in primary Sjögren’s syndrome and IgG4-RD

Primary Sjögren’s syndrome increases the risk of lymphoma development due to lymphatic organ involvement, especially B cells. In pSS, all elements of the immune response leading to the stimulation of maturation and activation of B lymphocytes and the disruption of the controlling mechanisms (breaching the control of, among others, T lymphocytes) result in the hyperreactivity of B cells.

Mucosa-associated lymphoid tissue (MALT) lymphoma is the most often observed lymphoma emerging during a course of pSS. Its different types are distinguished depending on their localization: gut-associated lymphoid tissue (GALT), bronchus-associated lymphoid tissue (BALT), or nasal-associated lymphoid tissue (NALT) lymphoma. Salivary gland MALT lymphoma is the most frequent localization of the initial lymphoproliferation in pSS (64, 65).

The main risk factors for lymphoma development in pSS are presented in **Table 5** (65).

**Table 5:** The risk factors for lymphoma development in pSS. Risk factors confirmed by Fragkioudaki et al.’s study (65) are in blue.

Domain	Feature/parameter
Clinical	Longer duration of pSS (longer time of mainly B cells but also T cells or NK cells stimulation)
	Persistent enlargement of salivary glands
	Lymphadenopathy
	Cryoglobulinemic vasculitis
	Palpable purpura
	Glomerulonephritis
	Raynaud ’s phenomenon
	Peripheral neuropathy
	Concurrent chronic infections (*Hepatitis C virus, Helicobacter pylori, Campylobacter jejuni, Borrelia burgdoferi, Chlamydophila psittaci*)
	Moderate/high-disease activity, calculated with ESSDAI or clinESSDAI
Serological	Leukopenia
	Low C4 component of complement
	Monoclonal gammopathy
	Cryoglobulinemia
	Autoantibody positivity (anti-SSA/Ro, anti-SSB/La)
	Rheumatoid factor
	FLT3L*
Histopathological	High focus score (≥3)
	Presence of germinal centers

*Fms-like tyrosine kinase 3 ligand (Flt-3L).

Risk factors for lymphoma development include Fms-like tyrosine kinase 3 ligand (Flt-3L), a type I transmembrane protein activating Flt-3 which stimulates progenitor cells in bone marrow and blood. A study revealed that high levels of Flt-3L were associated with lymphoma (66). The Flt3/Flt3L cascade is responsible for the development and maintenance of DCs (67). Flt3L – IgG4 (modified Fc region) has been investigated as a drug in immunotherapy and adjuvant in vaccines and may potentially become a drug for autoimmune diseases (68).

Lymphomas such as MALT lymphoma and diffuse large B cell lymphoma (DLBCL) can also develop in IgG4-related sialadenitis and orbital IgG4-RD localization (69).

A picture of salivary glands in ultrasonography with a high SGUS score evaluation can be a diagnostic predictor of risk of lymphoma development, particularly when it coincides with a high minor salivary gland biopsy (MSGB) focus score (70). Although this imaging method is not included in pSS classification criteria, its results may suggest a need for further lymphoma diagnostics. Another useful method, which can be used as a second step to better establish a place suspected of tumor development, infiltration, and lymphoproliferation, is magnetic resonance imaging (MRI) (71).

**Figure 2:**
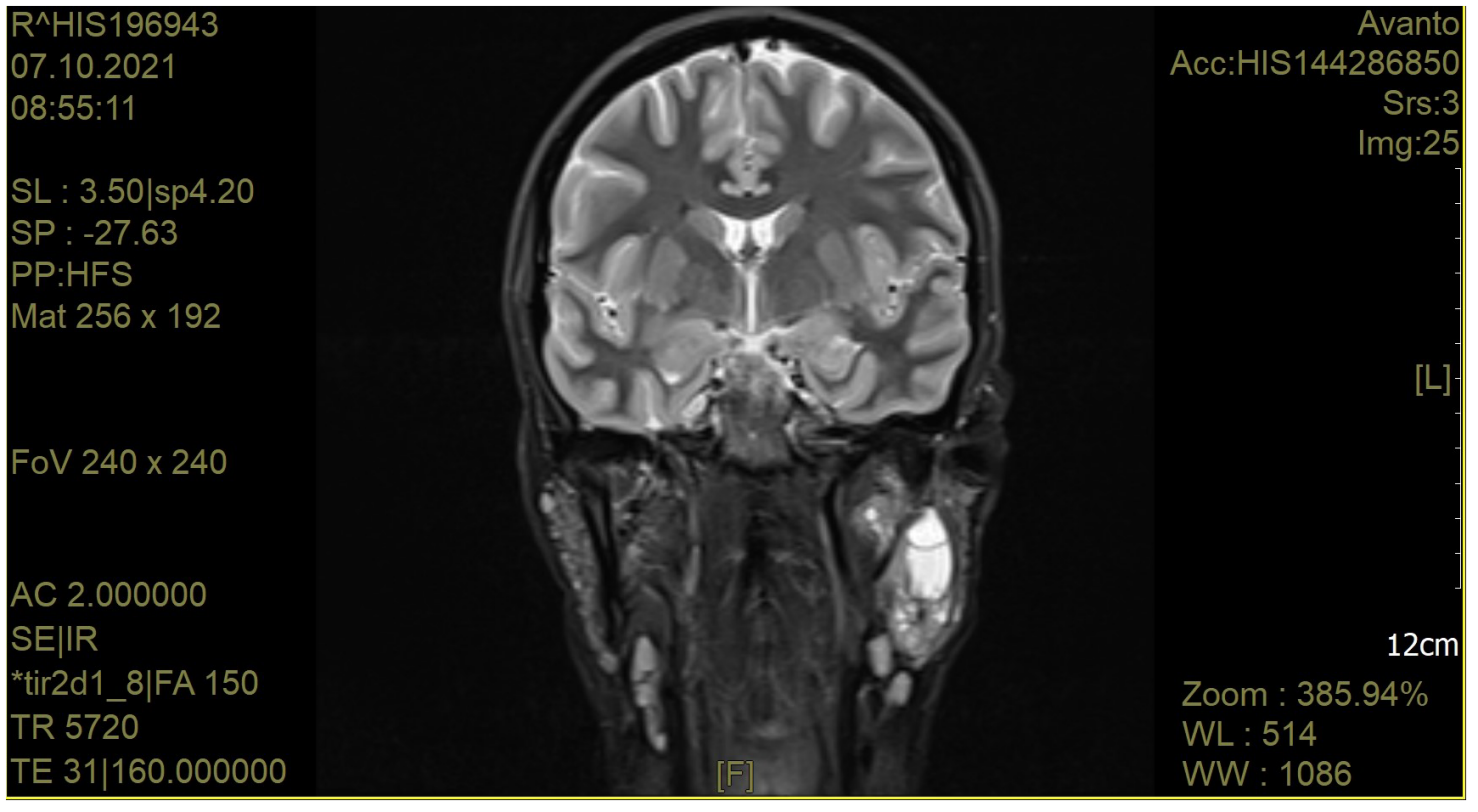
A craniofacial magnetic resonance image of a patient with pSS. The left parotid gland is bulging outwards. The right parotid gland is not enlarged. Altered vesicular structure of the parotid and submandibular glands. In the left parotid gland in the deep lobe, cystic lesions are visible. A group of cystic structures is visible in the central part of the left parotid gland. After i. v. contrast administration, no significant signal amplification was observed. Currently, the salivary ducts are not dilated. There are numerous lymphatic nodules in the parotid glands (approx. 6 mm). In the submandibular area, there are single lymph nodes up to 11 mm long. There are numerous lymph nodes in the neck, measuring up to 15 mm.

A craniofacial MRI image from a patient with pSS is shown in **Figure 2**.

IgG4-related sialadenitis is quite often present in this disease and may suggest pSS, particularly in the presence of changes in the major salivary glands. In such instances, the IgG4 concentration in the serum should be determined and an analysis of cells from a biopsy of the minor salivary glands performed. An examination of the material from a biopsy of the major salivary glands is useful both for establishing a diagnosis of IgG4-RD and verifying whether lymphoproliferation occurred.

Pulmonary nodular lymphoid hyperplasia (PNLH, pulmonary pseudolymphoma) is characterized by an increase in IgG4+ plasma cells and a higher IgG4+/IgG+ plasma cell ratio compared to other pulmonary lymphoid proliferations. In contrast, in a low-grade B cell lymphoma and BALT lymphoma, which may be associated with pSS, such a phenomenon was not confirmed (72, 73).

The presence of pSS, as a preexisting autoimmune disorder, was also reported in cases of bronchial-associated lymphoid tissue lymphoma (a relatively rare disease) (74, 75).

There are also various reports of an increased risk of carcinogenesis in IgG4-RD. In Yu et al.’s (76) metanalysis of 10 studies, the overall standardized incidence ratio (SIR) estimated an increased risk of overall cancer in IgG4-RD patients (SIR 2.57 95% CI 1.72–3.84) compared with the general population. This risk was particularly high in cases of pancreatic cancer and lymphoma (SIR 4.07 95% CI 1.04–15.92, SIR 69.17 95% CI 3.91–1223.04, respectively) (76).

In IgG4-RD the main risk factors for malignancy development are currently considered to be (77):

- autoimmune pancreatitis
- eosinophilia

However, the data about eosinophilia are conflicting. In some reports, it is presented as a protective factor (78) while in others, as a risk for cancer development (77).

IgG4-RD lymphadenopathy in some reports was misdiagnosed as Hodgkin’s lymphoma (79) but most reported cases indicate that MALT lymphomas more frequently occur in orbital IgG4-RD. The majority of these reports are derived from Asia (76). Further studies and meta-analyses should be performed to reliably assess the actual situation.

Taking into account that the possibility of lymphoma development exists both in pSS and IgG4-RD, but is higher in pSS, the differentiation between these two diseases may, in some especially serologically unclear cases, be crucial for diagnosis, proper treatment, and estimating the risk of lymphoma.

## A comparison of the main clinical features of primary Sjogren’s syndrome and IgG4-related disease

A summary of the main features of pSS and IgG4-RD is presented in **Table 6**.

**Table 6:** Comparison of the selected parameters/features of pSS and IgG4-RD.

Parameter/feature	pSS	IgG4-RD
Median age at diagnosis	>50*	50-60
Sex	F>M	M >F
Autoantibodies/immunological biomarkers	Anti-Ro antibodies, anti-La antibodies, ANA, RF,	none
IgG4 serum concentration	Normal or decreased	Usually elevated
Hypergammaglobulinemia**		
Mouth/eye dryness	Usually present	May be present
Salivary glands enlargement	Present (risk of MALT lymphoma)	Present (IgG4 related sialadenitis)
Lymphadenopathy	+	+
Cryoglobulins***	Mixed cryoglobulins usually present	Mixed cryoglobulins rarely present
Kidney	Tubulointerstitial nephritis, membranous nephropathy, renal tubular acidosis	Tubulointerstitial nephritis, membranous nephropathy
Retroperitoneum	is not typical, cases of idiopathic retroperitoneal fibrosis (IRF) were described	renal insufficiency due to a retroperitoneal fibrosis, typical periaortitis
Thyroid glands	Hashimoto’s disease	Riedel’s thyroiditis
Lungs	Interstitial lung disease inflammatory pseudotumor, interstitial pneumonitis, organizing pneumonia, lymphomatoid granulomatosis	Interstitial lung disease: nonspecific interstitial pneumonia, usual interstitial pneumonia lymphocytic interstitial pneumonia, organizing pneumonia
Gastrointestinal	Primary biliary cholangitis (PBC)	AIP type 1
Malignancy	Mainly lymphomas	Higher overall risk of malignancies in comparison to the general population (pancreatic; lymphoma)
Malignancy mimicker****	+	+

*currently, as a result of improved diagnostics and more widespread knowledge of pSS, it is often diagnosed at an earlier stage and in the population younger than >50 years.

**hypergammaglobulinemia.

***In both diseases mixed cryoglobulins can be present, as combination of monoclonal RF IgM and polyclonal IgG (type II) or combination of polyclonal RF IgM and polyclonal IgG (type III).

****Taking into account the clinical course of IgG4-RD with the formation of pseudotumors, organ and extra-organ infiltration (retroperitoneal fibrosis, periorbital infiltration, etc.), it seems that IgG4-RD may more often imitate malignancy, e.g. occurring as a pancreatic tumor in AIP-1.

AIP-1, autoimmune pancreatitis type 1; ANA, antinuclear antibodies; MALT, mucosa-associated lymphoid tissue; PBC, primary biliary cholangitis; RF, rheumatoid factor. + presence/confirmation.

When analyzing organ involvement in pSS compared to IgG4-RD, similarities and tropism of certain organs, e.g., salivary glands, in both diseases should be taken into account. Certain locations and types of lesions may be more indicative of one of these diseases. Thus, changes only in submandibular glands (e.g., Kuttner’s tumor, defined as a chronic sclerosing sialadenitis) without parotid gland involvement is a common feature of IgG4-RD and is rarely seen in pSS (8, 80).

Based on the studies indicating a reduction in IgG4 in the serum of patients with pSS (60), in the absence of specific autoantibodies, the assessment of IgG4 concentration may prove vital for the differentiation between pSS and IgG4-RD, with an increased concentration pointing to IgG4-RD and a decreased one indicating pSS.

Despite adopting the presence of several diseases as an exclusion criterium for the IgG4-RD diagnosis, there is an ongoing discussion on the potential possibility of IgG4-RD overlapping or co-existing with other diseases (81, 82). Addressing this problem requires further research and data gathering, which exceed the scope of this work.

## Conclusions

The main difference between IgG4-RD and pSS in the context of the presence of IgG4 is that while the serum concentration of IgG4 in IgG4-RD is typically elevated, the same phenomenon does not occur in pSS and some studies indicate a decrease in IgG4 serum level in pSS below its normal range.

This phenomenon may have a diagnostic value—in particular in the context of similar clinical features and affected organs in pSS and IgG4-RD—but its significance is not clear and this issue requires more research.

The role of IgG4 in pSS, as well as in IgG4-RD, is not fully understood and should be a subject of a further research. Currently, the still incomplete understanding of the dual role IgG4 plays in the immune response undermines the proper interpretation of the obtained test results.
